# Bibliographical

**Published:** 1889-04

**Authors:** 


### Bibliographical.

The volume of transactions of the joint meeting of the American and Southern Dental Associations held at Louisville, in August last, is at hand. It contains reports and papers of great value and interest; matter that should be in the hands of every dentist who desires to keep up with the times. It is always expected, that those who bring papers for these occasions will present the freshest thought on the subjects upon which they write. Every dentist's library should have the transactions of our various dental societies complete. Nowhere else will the matter be found that is contained in these books.

The volume contains 286 pages and is neatly gotten up. Those not members of the association can obtain the book of Dr. Geo. H. Cushing, Secretary of the American Dental Association, Chicago, at about the cost of publication.

A Healthy Body.—*A text book on Anatomy, Physiology, Hygiene, Alcohol and Narcotics; for use in intermediate grades in public and private schools, by Charles H. Stowell, M.D., Professor of Histology and Microscopy University of Michigan; Author of Students Manual of Histology; The Human Tooth, &c., &c., fully illustrated with original sketches by the author.*

This little work, Elementary Physiology and Hygiene, is the best presentation of this, or rather, these subjects, that we have seen; almost every thing pertaining to the subject is embraced, and yet so clearly stated that any child who can read can understand it. It is a marvel of condensation; that so much can be said in so few words will be a surprise to every one who looks over it.

Professor Stowell's life-work has eminently fitted him for the preparation of such a work as this; and this is one of his successes, the out-reach of which can not be measured, nor approximately anticipated. This book ought to be in every school in the land, and in the hands of every child who can read it. It will be of immense value to teachers as well, as it will enable them to most profitably present these subjects to their pupils. The work will doubtless be obtainable of all booksellers; indeed it ought to be wherever school books are sold. It is published by John C. Buckbee, Chicago.

